# Structure Analysis of a New Psychrophilic Marine Protease

**DOI:** 10.1371/journal.pone.0026939

**Published:** 2011-11-23

**Authors:** Si-Cai Zhang, Mi Sun, Tang Li, Qi-Hai Wang, Jian-Hua Hao, Yi Han, Xiao-Jian Hu, Ming Zhou, Sheng-Xiang Lin

**Affiliations:** 1 Laboratory of Structural Biology, Institute of Biochemistry and Cell Biology (IBCB), Shanghai Institutes of Biological Sciences (SIBS), Chinese Academy of Sciences, Shanghai, China; 2 Yellow Sea Fishery Research Institute, Chinese Academy of Fisheries Science, Qingdao, Shandong, China; 3 Laboratory of Oncology and Molecular Endocrinology, CHUL Research Center (CHUQ) and Laval University, Quebec, Canada; King's College, London, United Kingdom

## Abstract

A new psychrophilic marine protease was found from a marine bacterium *Flavobacterium YS-80* in the Chinese Yellow Sea. The protease is about 49 kD with an isoelectric point about 4.5. It consists of 480 amino acids and is homologous to a psychrophilic alkaline protease (PAP) from an Antarctic Pseudomonas species. The protein was purified from the natural bacterium fermented and crystallized. Its crystal structure (PDB ID 3U1R) was solved at 2.0 Å by Molecular Replacement using a model based on PAP, and was refined to a crystallographic R_work_ of 0.16 and an R_free_ of 0.21. The marine protease consists of a two domain structure with an N-terminal domain including residues 37–264 and a C-terminal domain including residues 265–480. Similar to PAP, the N-terminal domain is responsible for proteolysis and the C-terminal is for stability. His186, His190, His196 and Tyr226 are ligands for the Zn^2+^ ion in the catalytic center. The enzyme's Tyr226 is closer to the Zn^2+^ ion than in PAP and it shows a stronger Zn^2+^―Tyr-OH bond. There are eight calcium ions in the marine protease molecule and they have significantly shorter bond distances to their ligands compared to their counterparts in all three crystal forms of PAP. On the other hand, the loops in the marine protease are more compact than in PAP. This makes the total structure stable and less flexible, resulting in higher thermo stability. These properties are consistent with the respective environments of the proteases. The structural analysis of this new marine protease provides new information for the study of psychrophilic proteases and is helpful for elucidating the structure-environment adaptation of these enzymes.

## Introduction

**Figure 1:**
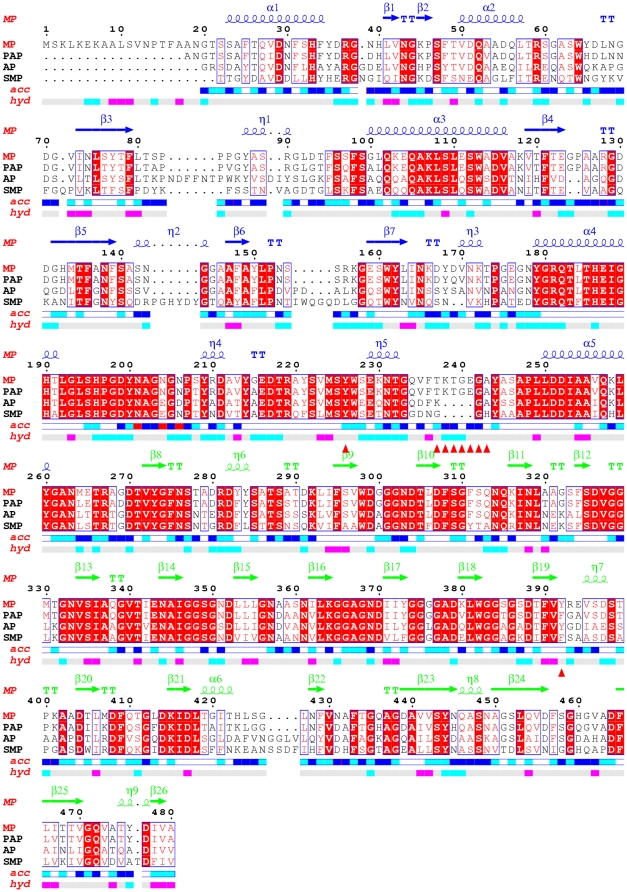
Sequence alignment of MP, PAP, AP and SMP. Secondary structure elements for MP are indicated in blue for the proteolytic domain and in green for the C-terminal domain. The relative accessibility of each residue rendered as blue-colored boxes from dark blue (accessible) to white (buried residues). The hydropathic character of the sequence calculated according to the algorithm of Kyte and Doolittle with a window of 3 also is shown, with the color varying from pink (hydrophobic) through grey (intermediate) to cyan (hydrophilic). Further, Tyr226 of the active site, loop 237–243 and residue 392 are marked as red triangles.

Many extreme microbes have been found on the seabed. These organisms live at temperatures close to freezing point. To survive, the organisms' enzymes have evolved to adapt to the cold environment.[1]–[5] The psychrophilic alkaline protease is one of these enzymes. To date, several alkaline proteases have been found in different locations and have been crystallized, including the psychrophilic enzymes and their mesophilic counterparts. Structural studies of these proteases are important to elucidate the structure-environment adaptation of proteins. They also provide useful information for industrial utilization of their functions such as developing efficient detergents. A new psychrophilic marine protease (MP) has been found from a marine bacterium *Flavobacterium YS-80* which was recently isolated from the Yellow Sea in China. MP consists of 480 amino acids, is homologous to psychrophilic alkaline protease (PAP, from an Antarctic Pseudomonas species), a mesophilic counterpart alkaline protease (AP, from P. *aeruginosa*) and serralysin (SMP, from S. *marcescens*) according to sequence alignment (Fig. 1). It also belongs to the serralysin family comprising a group of bacterial metalloproteases.[6]–[9] The MP displays 88% sequence identity with PAP, and 60% with AP. This protease is a secreted protein that has a molecular mass of 49320 Daltons and an isoelectric point of about 4.5. Studies have shown that the preferred temperature for its catalyst activity is 30°C, higher than the 20°C for PAP (Table 1). PAP exhibits maximum activity at pH 9.5 and its activity is strongly inhibited by EDTA.[8], [10] To date three forms of PAP structures (PDB ID 1H71, 1G9K and 1OMJ) have been obtained under different crystallization conditions, which share almost the same structure with the exception of small conformational differences in several amino acids and metal ion quantity.[9], [11] The crystal structure of AP was initially determined by a complex of AP with a mixture of tetrapeptide products.[12] Then the crystal structure of unliganded AP (PDB ID 1AKL) from P. *aeruginosa* was determined at 2.0 Å by X-ray method.[7]

**Table 1:** Psychrophilic character of PAP, MP and AP.

Alkaline proteases	Psychrophilic nature	Optimal catalytic temperature	Preferred pH For catalysis	Sequence Identity
PAP	Most psychrophilic	20°C	9.5	0.88
MP	psychrophilic	30°C	8–11	1
AP	mesophilic	60°C	7–9	0.6

Similar to PAP, MP is also a cold adapted protease. Compared with the mesophilic proteins, these proteins are usually more flexible and have looser surface loops and weaker hydrogen bonding.[4] Although PAP and MP are both psychrophilic protease, the difference of their living environments, for example the temperature, may explain some differences in their biochemical properties and structure.

Here we report the crystallization and structural analysis of native MP. The structural differences between MP and various forms of PAP and AP have been compared.

## Results and Discussion

### Inductive Coupled Plasma Emission Spectrometer (ICP-AES) analysis

Four kinds of atoms were detected in the marine protease by the ICP analysis. In order to avoid errors resulting from contaminant ions, the sample buffer used to dissolve the protein was adjusted to 10 mM Tris pH 8.0 with 100 mM NaCl by repeated dilution and concentration. Calcium, zinc and phosphate could be confirmed in the marine protease. According to the data from ICP analysis, the molarity of calcium was about eight times that of zinc (Table 2). This information is consistent with the real ratio of calcium to zinc in the MP protein.

**Table 2:** ICP Analysis for Marine Protease.

atom	Mean (mg/L)
Ca	1.805
Zn	0.381
Na	16.091
P	0.693

### Overall structure

The MP crystal structure (PDB ID 3U1R) with 456 amino acid residues was solved by molecular replacement using the structure of PAP form 3 (PDB ID 1OMJ) as a model, and refined to 2.0 Å resolution with an R_work_ of 0.16 and an R_free_ of 0.21. The refinement statistics are given in Table 3. One Zn^2+^ ion and 8 Ca^2+^ ions have been located in the electron density map. The MP has a similar overall structure to PAP, AP and SMP with an N-terminal domain comprised of residues 37–264 and a C-terminal domain containing residues 265–480.[9] The N-terminal domain is the proteolytic domain which contains the active zinc ligation. The latter consists of a parallel β-roll structure and seven bound Ca^2+^ ions and is mainly responsible for the stability of the protease.

**Table 3:** Crystallographic Data for Marine Protease.

Space group	P2_1_2_1_2_1_
Unit cell parameter (Å)	a = 45.91, b = 75.20,c = 115.42
No. molecules (a.u.)	1
Matthews Coefficient (Å^3^/Da)	1.96
Data collection	
Resolution (Å)	50–2.00 (2.05–2.00)*
No. reflections used	28189
Completeness (%)	91.99 (63.44)
Rmerge (%)	7.3 (16.6)
I/sig (I)	17.9 (4.2)
Redundancy	5.6 (4.9)
Model and refinement statistics	
Protein (456 residues)	3568
Water	166
Metal ions	9
R_work_ (%)	16.14
R_free_ (%)	21.15
Reflections included in R_free_ set	1304
Average B-factor (Å^2^)	
Main-chain	25.11
Side-chain	26.43
Water molecules	30.88
Ions	28.02
Stereochemical parameter	
Bond length	0.018Å
Bond angle	1.6°
Ramachandran plot analysis	
Most favorable regions	92.9%
Additionally allowed regions	7.1%

*Parameter in highest shell.

**Figure 2:**
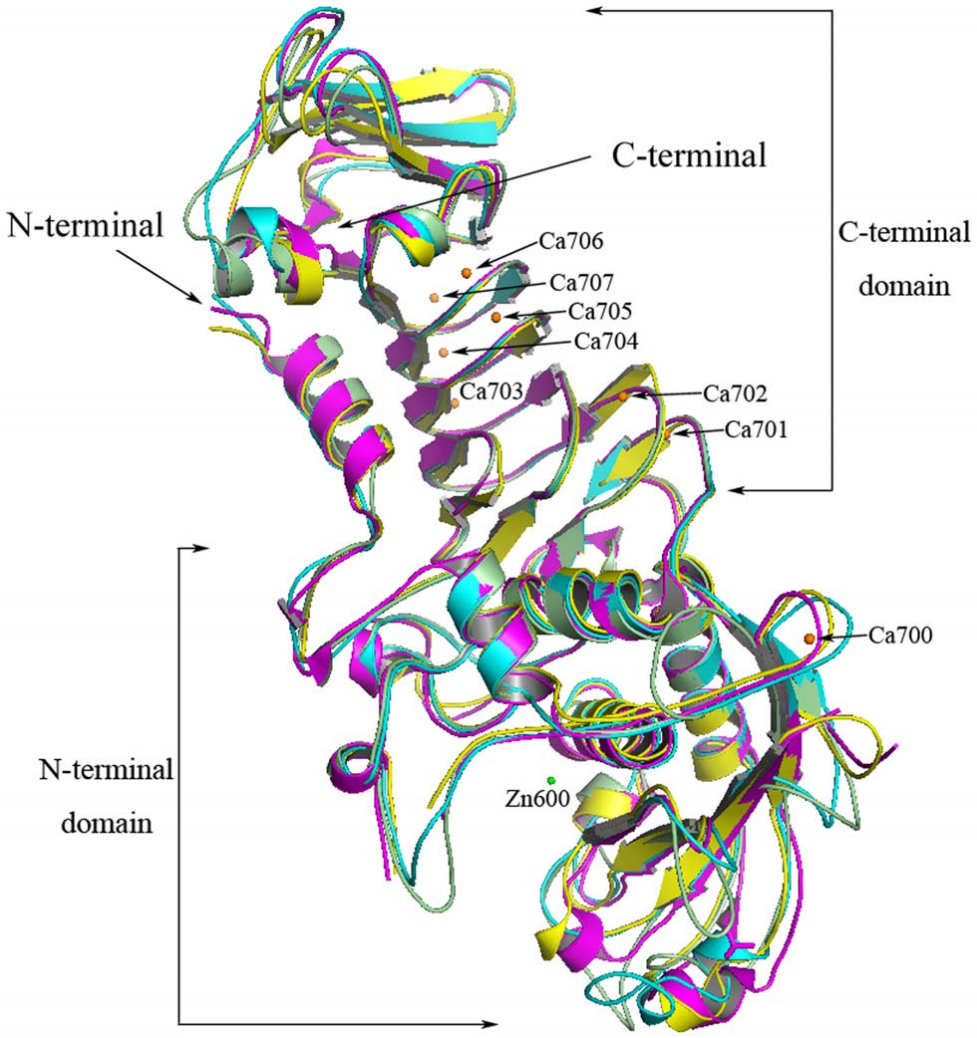
Superposition of MP (yellow) with PAP form 2 (magenta) and AP (cyan) and SMP (pale green). Only the Zn and Ca ions from the MP structure are shown in the figure.

After superposition of the overall structure of MP with PAP, AP and SMP using all the C_α_ atoms, the main-chain RMSDs were 0.87 Å (with PAP form1), 1.08 Å (with PAP form2), 1.03 Å (with PAP form3), 1.00 Å (with AP) and 1.28 Å (with SMP) (Fig. 2). The MP and PAP differ by 42 amino acids, of which 35 are conserved. Most of the different residues are located on the surface and 27 residues are in the C-terminal domain. These different residues are not involved in the catalytically active region. Interestingly residue Tyr392, the counterpart of Phe375 in PAP, forms an additional hydrogen bond with Asp404 helping to stabilize the beta barrel structure (Fig. S1).

### Zinc binding active site

**Figure 3:**
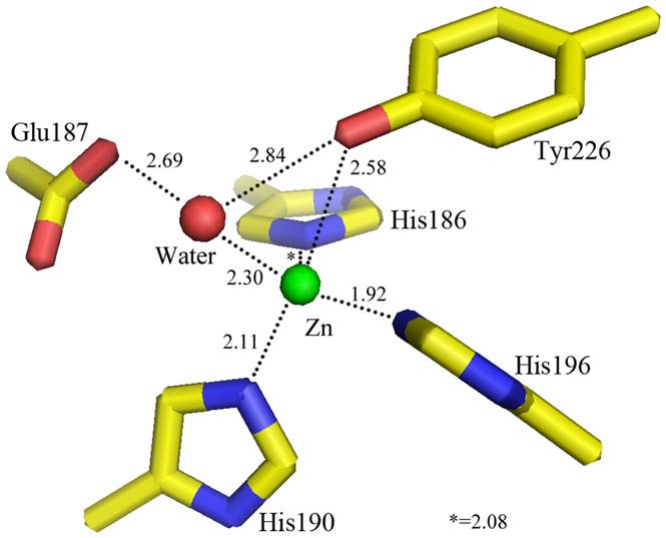
Active site environment showed the Zn atom (green) coordinating to His186, His190, His196, Tyr226 and one water molecular (red). The number besides the dash line shows the interaction length.

The classic sequence motif HEXXHXXGXXHZ (residues 186–197 in MP) for “metzincins” is conserved in the marine protease,[13] in which His186, His190, His196 and Tyr226 are ligands for the Zn^2+^ ion. Among the 3 forms of the PAP crystals, only form 1 has Tyr209 (Tyr226 in MP) bound to the Zn^2+^ ion.[11] Even so, the Tyr226 in MP is closer to the Zn^2+^ ion than in PAP (Table 4) and it shows a stronger Zn^2+^―Tyr-OH bond (Fig. 3). The distance between Tyr226 and Zn^2+^ ion is 2.58 Å in MP, but the distance is 2.94, 4.24 and 4.27 Å respectively in the form 1, form 2 and form 3 crystals for PAP. In AP, this distance is 3.02 Å. The bond difference in MP and PAP is significant, but that between MP and AP is less significant. In PAP, a weaker Zn^2+^―Tyr-OH bond was considered to facilitate the coordination of the substrate to the Zn^2+^ site for the psychrophilic protease. In other words, PAP has a more flexible activity center than MP. This likely maintains the activity of the psychrophilic protease at very low temperatures. The stronger bond may imply that the MP has less flexibility at the same low temperature compared to PAP. It means that the MP is more like AP than PAP at the catalytic site.

**Table 4:** Distance of ligands to the zinc ion at the active site in MP compared with PAPs and AP (Å).

Ligands (MP/PAP/AP)	MP	PAP (form1)	PAP (form2)	PAP (form3)	AP
His186/His169/His176	2.08	2.29	2.10	2.16	2.22
His190/His173/His180	2.11	2.24	2.01	2.23	2.20
His196/His173/His186	1.92	2.26	2.07	2.15	2.16
water	2.30	2.25	2.18	2.47	2.36
Tyr226/Tyr209/Tyr216	2.58	2.96	4.24	4.27	3.02

### Calcium ions

**Figure 4:**
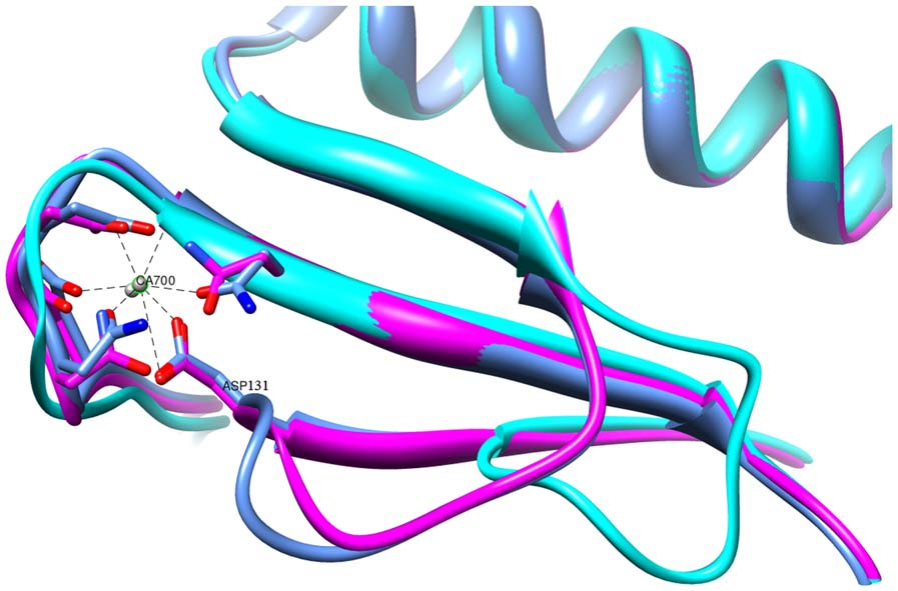
Superposition of MP (cornflower blue) with PAP form 1 (purple) and AP (cyan) shows the N-terminal additional Ca-binding site, indicating a stabilized loop conformation formed in MP. The MP amino acids coordinated to the Ca ion are shown as sticks.

In total, there are eight calcium ions in the MP molecule, eight, seven and eight in forms 1, 2 and 3 of the PAP crystals respectively and eight in AP. In contrast to MP and PAP, AP contains eight calcium ions which only exist in the C-terminal domain. In MP and PAP, the calcium ions occupy the same positions, with one in the N-terminal domain (the catalytic domain) and the others in the C-terminal domain. The additional calcium ion (Ca508) in the C-terminal domain of AP is coordinated by five amino acids (Asp463, Ser465, Asp467, His469, Asp471) in one loop on the surface of the protein, and this ligation makes the loop more compact. Compared to AP, the compartment loops in MP and PAP are more flexible. Among the ligands of the additional N-terminal calcium ion (Ca700) in MP and PAP, there is one amino acid (Asp131) that belongs to a loop which is significantly longer and more extended on the surface of protein than the corresponding loop in AP. If the calcium ion didn't exist, the loop would be too flexible, making the protein unstable (Fig. 4). Evidence also comes from the relative B-factors of Ca700 (Table 5), indicating that residues Asp66, Asn68, Asp 70, Val72, Asn74 and Asp 131 are ligands of Ca700.

**Table 5:** Relative B-factors for calcium ions.^†^

% B-factor in:				
MP	PAP form 1	PAP form 2	AP	SMP
(707)161	(501)82		(505)125	(478)74
(706)85	(504)86	(706)101	(507)91	(479)67
(705)89	(505)85	(705)97.5	(506)102	(476)53
(704)91	(502)78	(704)103	(504)107	(477)67
(703)92	(503)81	(703)86	(503)80	(475)66
(702)88	(506)83	(702)97	(502)90	(474)62
(701)83	(507)88	(701)83	(501)89	(473)71
(700)91	(500)128	(700)159		
			(508)226	

† The relative B-factor is obtained from B-factor dividing by the mean B-factor of the whole protein. Presented on the same line are the calcium ions from the respective structures that are spatially equivalent. The calcium numbering is given in brackets.

Among all the calcium ions in MP, only Ca707's position is replaced by a water molecule in PAP form 2. This calcium ion is the most unstable. It has the highest relative B-factor (Table 5) and has the longest average distance (Table 6). Of the eight calcium ions in MP, five (Ca703, Ca704, Ca705, Ca706 and Ca707) are bound in the core of the parallel β-roll. It is not easy for other ions to reach and occupy the position of ligands. In comparison to the other two calcium ions (Ca700 and Ca701) where all the coordination positions are occupied by amino acid residues, Ca702 has increased exposure to the solvent. This is consistent with the binding studies in crystal form 2 of PAP.[11] Here the zinc ion was the first metal ion chelated by increasing EDTA concentrations. Subsequent chelations were for Ca700 and then Ca702.

**Table 6:** Average Distance (Å) of calcium ions and its ligands in MP, PAP (all 3 forms), AP and SMP.†

Calcium	MP	PAP form 1	PAP form 2	PAP form 3	AP	SMP
Ca707	2.51	2.53	none	2.72	2.56	2.63
Ca706	2.33	2.48	2.37	2.71	2.36	2.53
Ca705	2.32	2.54	2.46	2.62	2.37	2.42
Ca704	2.39	2.54	2.43	2.55	2.46	2.42
Ca703	2.48	2.54	2.44	2.57	2.37	2.40
Ca702	2.42	2.53	2.44	2.67	2.43	2.35
Ca701	2.36	2.49	2.39	2.57	2.38	2.35
Ca700	2.35	2.78	2.33	2.64	3.04	none

† A cutoff distance of 3.2 Å has been used.

The distances between calcium ions and their ligands were calculated (Table 6). Results show that some calcium ions in MP, AP and SMP have shorter bond distances to their ligands than their counterparts in all three crystal forms of PAP. This indicates that the structure of PAP is more flexible than MP, AP and SMP to some extent. It is uncertain whether Gln410 is one of the ligands in the vicinity of Ca707 (MP), because of missing electron density in the Gln410 side-chain. But in the crystal form 1 of PAP, the counterpart amino acid of Gln393 is confirmed to be a ligand for the calcium ion (Ca501 in PAP form 1).

### Loops

**Figure 5:**
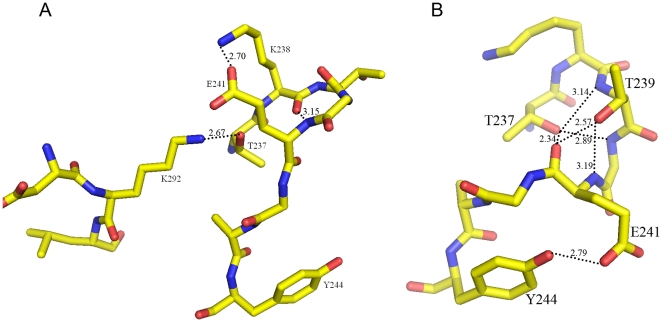
Conformations of the loops containing residues 220–226 in PAP form 2 (A) and counterpart residues of 237–243 in MP (B). Hydrogen bond is shown as a red line.

MP is more compact in overall structure than PAP but is less compact than AP. The main differences are caused by several loops which move toward the internal side of the protein. These loops include residues 38–46, 84–87, 237–243, 422–438, 445–449 and 457–464. In the loop containing residues 220–226 (counterpart of 237–243 in MP), PAP form 1 demonstrates a hairpin conformation with two hydrogen bonds in the main chain formed between Lys221 and Glu224. In PAP form 2, the hairpin structure is replaced by another conformation, which is stabilized by the hydrogen bonds between Thr220-Lys275 and Lys221-Glu224 (Fig. 5 A). PAP form 3 has only one hydrogen bond in this loop, resulting in an even more flexible conformation. Unlike the PAPs, MP exhibits a more compact structure, the C_α_ atom of Thr239 (Thr222 in PAP) moves about 9.96 Å toward the internal side of the protein. Hydrogen bonds are formed between Thr237, Thr239, Gly240 and Glu241. The side chains of Glu241 and Tyr244 also form a hydrogen bond which makes the loop more stable (Fig. 5 B). In AP and SMP, the loop counterpart is significantly shorter and this makes the whole protein compact. This is in agreement with the generally accepted concept that cold adapted proteins are more flexible with more elastic loops.

### Conclusion

PAP originates from a microorganism living in the Antarctic Ocean whereas MP came from the Yellow Sea in China (Pacific Ocean). AP is their thermo homologue. In general, psychrophilic enzymes have a longer loop, low proline and arginine contents and more glycine residues.[14], [15] MP has 2 glycines, 1 proline and 1 arginine more than PAP. Thus there is no significant difference in the content of these residues. This may be because MP and PAP are both psychrophilic enzymes that share similar characteristics in order to adapt to low environmental temperatures. However, they come from different oceans and bear different selective constraints due to temperature difference. During evolution, some differences in protein composition and structural displacements will occur with adaptation to environmental changes. For example, the Zn^2+^―Tyr-OH bond in PAP is more flexible in order to facilitate substrate accessibility and to maintain its activity in very low temperatures. MP contains seven more glycines than AP. Glycine confers flexibility to the protein thus suggesting that MP is more flexible than AP.[16]

The specific activity of MP decreases with gradual temperature increase. PAP is three times more active at 20°C than a mesophilic counterpart from Pseudomonas aeruginosa. At 45°C, PAP is rapidly inactivated. MP loses nearly 92% activity at 50°C at pH 8.0.[10] This suggests that MP has better thermo stability than PAP. These properties are also consistent with the environment where the proteases exist. The PAP from the Antarctic Ocean bears less selective stress at high temperatures than the MP from the Yellow Sea of the Pacific Ocean. In contrast, MP has adapted to the higher temperature of the Yellow Sea with better thermo stability than that of the Antarctic Ocean.

In general, MP has more thermo stability and is more rigid in structure than PAP but is more flexible than AP. This suggests that the structural differences between MP, PAP and AP are a consequence of their respective evolutionary processes in different environments requiring temperature adaptation.

## Materials and Methods

### Purification of Marine Protease

The protein was extracted from the marine bacterium *Flavobacterium YS-80* fermented in the lab. The bacterium was fermented at the optimal temperature (18–23°C) for up to 48 h, followed by centrifugation of the fermentation solution. The pellet was discarded. The marine protease was secreted into the culture medium. The supernatant was then concentrated by passage through a 10 kDa ultrafiltration membrane (Millipore) and the partially purified marine protease was lyophilized. The protein powder was then dissolved in 10 mM Tris pH 8.0, 100 mM NaCl buffer and further purified by Superdex 200 (GE) gelfiltration chromatography (Fig. S2).

### Inductive Coupled Plasma Emission Spectrometer (ICP-AES) analysis

Inductively coupled plasma atomic emission spectroscopy (ICP-AES) is an analytical technique that can be used to detect the metal atoms in proteins. The intensity of the emission spectroscopy is proportional to the concentration of metal atoms in the protein. In order to obtain more information about the protein, for example the heavy metal atom chelated by the protein, ICP analysis was conducted. The protein was prepared in a buffer of 10 mM Tris pH 8.0, 100 mM NaCl, and sent to the Shanghai Institute of Organic Chemistry, Chinese Academy of Sciences.

### Crystallization

The protein collected from the gel filtration chromatography was concentrated to about 20 mg/ml. Crystallization screen was carried out with hanging drop vapor diffusion at different temperatures. The protein was crystallized in the presence of 0.2 M sodium acetate, 0.1 M Tris, 30% PEG 4 K, pH 8.5 at 20°C. The preliminary crystals were needle shaped and could not be used for data collection. The crystallization was optimized and cubic crystals were obtained in the presence of 0.2 M sodium acetate, 0.1 M Tris, 25% PEG 4 K, 0.1 M Li_2_SO_4_, pH 8.0 at 4°C (Fig. S3). These crystals were packed into a capillary and sent to APS in Chicago where their diffractions were collected.

### Structure determination and refinement

The native protein data collection was carried out at a wavelength of 1.1000 Å for Zn ion absorption. The crystal diffracts to 1.8 Å with space group P2_1_2_1_2_1_. The data were processed and scaled by the software Denzo/SCALEPACK.[17] The structure of PAP form 3 (PDB ID 1OMJ) served as a model in a molecular replacement search conducted with the program MolRep.[18] The structure was refined with manual adjustment using Coot[19] and Refmac5[20]. An anomalous difference Fourier map was calculated by using the program CNS[21] to confirm the presence or absence of all the ions, where *F*_calc_ comes from a refined model in which metal ions have been depleted. Because of poor or missing electron density, residues 1–19 and 201–205 were not inserted into the electron density map as for the PAP protein structure. Moreover side chain residues for Glu104, Arg128, Lys172, Arg210, Lys230, Lys238, Glu265 were not observed in the electron density map. The atomic coordinates and structure factors have been deposited in the Protein Data Bank, www.pdb.org (PDB ID 3U1R). Model qualities were checked with PROCHECK[22] and WHATCHECK[23]. The figures was plotted with ESPript[24] and PyMOL[25].

## Supporting Information

Figure S1 **Ca706 binding site in MP. This shows the ligand Asp404 stabilized by Tyr392 through hydrogen bond.**
(TIF)

Figure S2 **SDS-PAGE gel shows the MP protein purified after gelfiltration column (Supdex 200).**
(TIF)

Figure S3 **(a) Preliminary crystal of marine protease obtained using the matrix screening approach.** The crystal was grown at 20°C. **(b) The optimized crystal of marine protease.** Compared to the previous crystallization, Li_2_SO_4_ was added into the well solution and the PEG 4K concentration was decreased to 25%. The crystal was grown at 4°C.
(TIF)
