# Supplementary figures and images for: Structure Analysis of a New Psychrophilic Marine Protease
Source: PLoS One. 2011 Nov 23;6(11):e26939. doi: 10.1371/journal.pone.0026939 (PMC3223159; doi:10.1371/journal.pone.0026939)

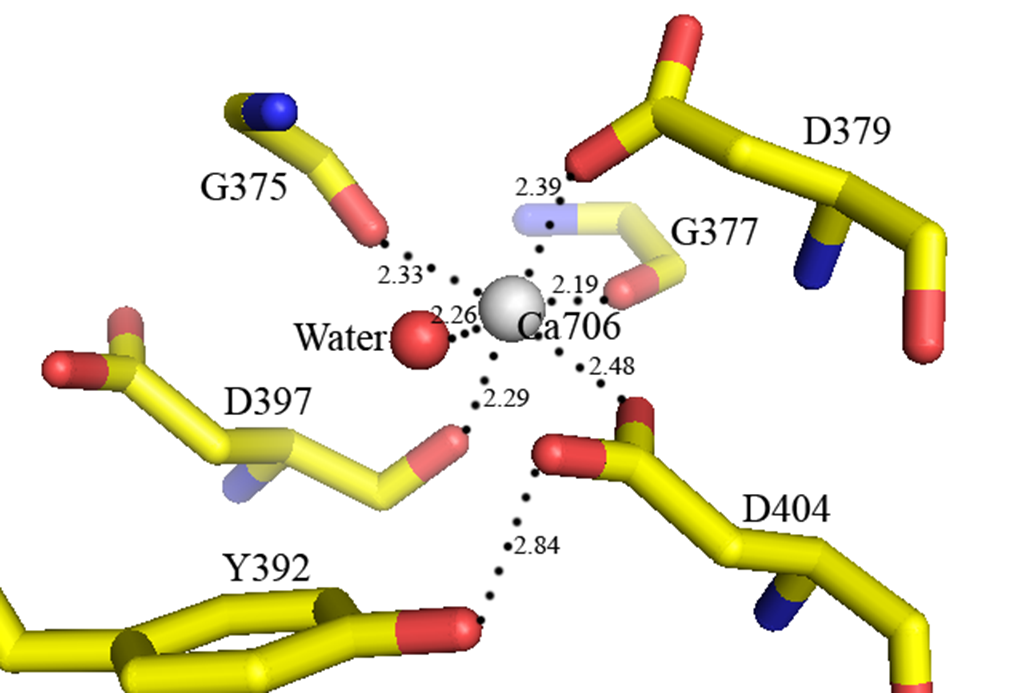

Supplement: Figure S1 — Ca706 binding site in MP. This shows the ligand Asp404 stabilized by Tyr392 through hydrogen bond. (TIF) [file pone.0026939.s001.tif]

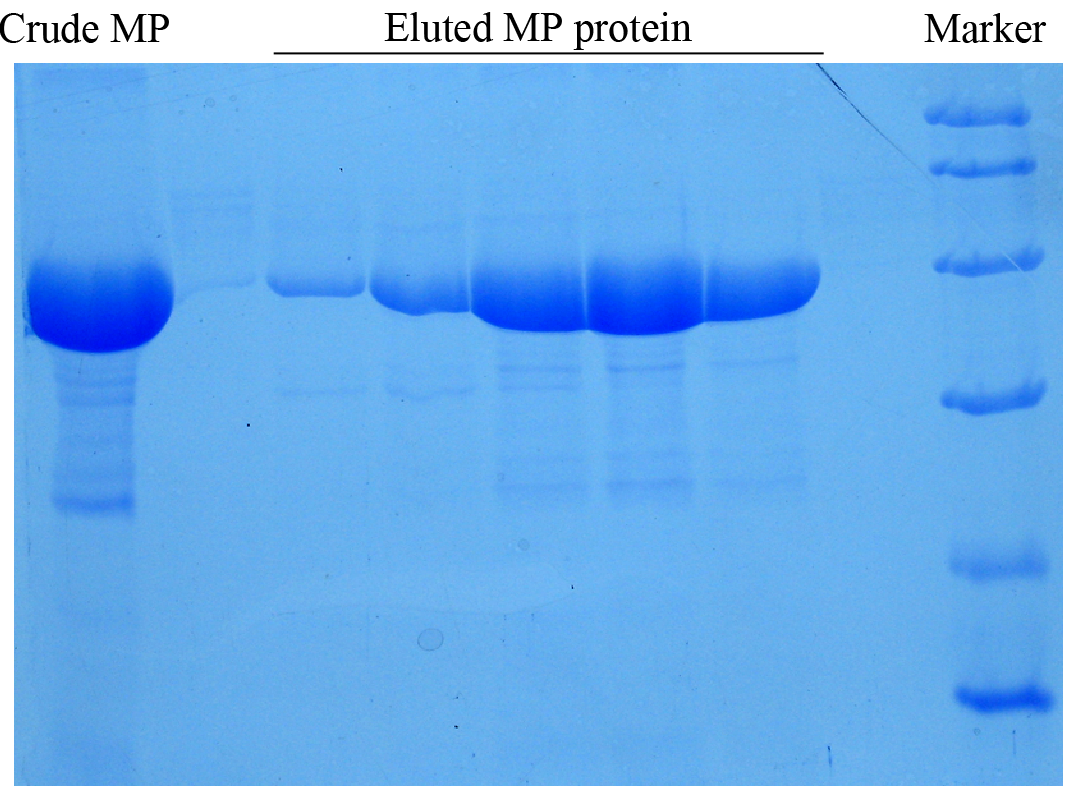

Supplement: Figure S2 — SDS-PAGE gel shows the MP protein purified after gelfiltration column (Supdex 200). (TIF) [file pone.0026939.s002.tif]

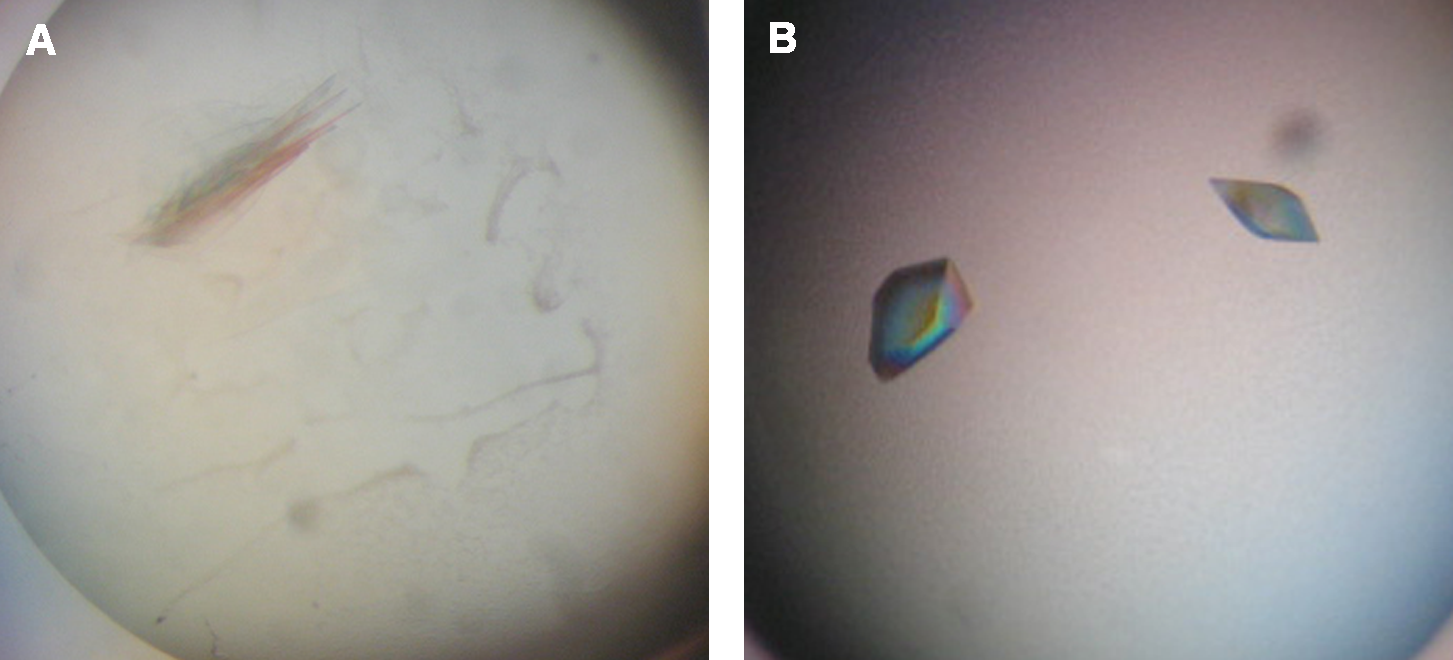

Supplement: Figure S3 — (a) Preliminary crystal of marine protease obtained using the matrix screening approach. The crystal was grown at 20°C. (b) The optimized crystal of marine protease. Compared to the previous crystallization, Li2SO4 was added into the well solution and the PEG 4K concentration was decreased to 25%. The crystal was grown at 4°C. (TIF) [file pone.0026939.s003.tif]
